# Circular of the Committee of Publication of Am. Med. Association

**Published:** 1851-09

**Authors:** 


*Circular of the Committee of Publication of Am. Med. Association.*—We have received a printed circular from the Com. of Publication of the Am. Med. Association, stating that the estimated cost of the publication of vol. IV. is about sixteen hundred dollars, while the funds in the treasury amount to but about one thousand dollars.

This excess of expenditure arises from the cost of publishing the prize essay of Prof. Dalton, amounting to about seven hundred dollars. The publication of this essay in the Transactions was ordered by the Association. The Committee, of which Isaac Hays, M. D., is chairman, state that Prof. Dalton's essay "is a paper of great merit, exhibiting extensive research, and is illustrated with numerous beautiful drawings, a number of them colored. The publication of this essay will do credit to the Association, and tend to elevate the scientific character of the American Medical Profession." The Committee, therefore, appeal to members of the Association to make up the requisite sum to cover the whole cost of issuing the volume of Transactions for the present year. And they propose the following plan: "A large number of copies of the three volumes already published remain unsold, and, if the members will complete their sets, and use their influence to extend the sale of these volumes, the required sum may be easily raised."

The published volumes are now furnished to members, or to societies, which have been represented in the Association, at the following rates:

| | |
|---|---|
| Either of the first three volumes separately (in paper covers) | $1 50 |
| A complete set in three volumes (paper covers) - - | 4 00 |
| Do. do. do. (cloth) - - - | 5 00 |
| Single copies of vol. iv. (to permanent members) - - | 2 00 |
| Three do. do. do. do. - - | 5 00 |

In all cases, the amount must be remitted to the Treasurer of the Association, Isaac Hays, M. D., Philadelphia.

We would urge upon our readers the favorable consideration of the above appeal, and the necessity of prompt action in order that the publication of the volume of Transactions may not be delayed.

